# Inhibition of Cyclooxygenase-2 Reduces Hypothalamic Excitation in Rats with Adriamycin-Induced Heart Failure

**DOI:** 10.1371/journal.pone.0048771

**Published:** 2012-11-13

**Authors:** Min Zheng, Yu-Ming Kang, Wei Liu, Wei-Jin Zang, Cui-Yu Bao, Da-Nian Qin

**Affiliations:** 1 Department of Physiology, Shantou University Medical College, Shantou, P. R. China; 2 Department of Biomedical Engineering, Hubei University of Science and Techonology, Xianning, P. R. China; 3 Department of Physiology and Pathophysiology, Xi’an Jiaotong University School of Medicine, Xi’an Jiaotong University Cardiovascular Research Center, Xi’an, P. R. China; 4 Department of Pharmacology, Xi'an Jiaotong University School of Medicine, Xi’an, P. R. China; University Hospital of Würzburg, Germany

## Abstract

**Background:**

The paraventricular nucleus (PVN) of the hypothalamus plays an important role in the progression of heart failure (HF). We investigated whether cyclooxygenase-2 (COX-2) inhibition in the PVN attenuates the activities of sympathetic nervous system (SNS) and renin-angiotensin system (RAS) in rats with adriamycin-induced heart failure.

**Methodology/Principal Finding:**

Heart failure was induced by intraperitoneal injection of adriamycin over a period of 2 weeks (cumulative dose of 15 mg/kg). On day 19, rats received intragastric administration daily with either COX-2 inhibitor celecoxib (CLB) or normal saline. Treatment with CLB reduced mortality and attenuated both myocardial atrophy and pulmonary congestion in HF rats. Compared with the HF rats, ventricle to body weight (VW/BW) and lung to body weight (LW/BW) ratios, heart rate (HR), left ventricular end-diastolic pressure (LVEDP), left ventricular peak systolic pressure (LVPSP) and maximum rate of change in left ventricular pressure (LV±dp/dtmax) were improved in HF+CLB rats. Angiotensin II (ANG II), norepinephrine (NE), COX-2 and glutamate (Glu) in the PVN were increased in HF rats. HF rats had higher levels of ANG II and NE in plasma, higher level of ANG II in myocardium, and lower levels of ANP in plasma and myocardium. Treatment with CLB attenuated these HF-induced changes. HF rats had more COX-2-positive neurons and more corticotropin releasing hormone (CRH) positive neurons in the PVN than did control rats. Treatment with CLB decreased COX-2-positive neurons and CRH positive neurons in the PVN of HF rats.

**Conclusions:**

These results suggest that PVN COX-2 may be an intermediary step for PVN neuronal activation and excitatory neurotransmitter release, which further contributes to sympathoexcitation and RAS activation in adriamycin-induced heart failure. Treatment with COX-2 inhibitor attenuates sympathoexcitation and RAS activation in adriamycin-induced heart failure.

## Introduction

Congestive heart failure (HF) is a serious cardiovascular disease that increases morbidity and mortality and causes an economic burden on families and societies. Unfortunately, the mechanism of HF is not clear. Increased sympathetic drive is one of the pathophysiological characteristics of HF, and it is a major contributor to the morbidity and mortality of HF patients. Recently, researchers demonstrated that a central nervous system mechanism contributes to the sympathetic nervous system (SNS) abnormality in HF [1]–[3]. The paraventricular nucleus (PVN) of hypothalamus is an important center for the integration of sympathetic nerve activity [4] and the regulation of cardiovascular function and fluid homeostasis [5].

Large amounts of excitatory and inhibitory neurotransmitters, such as glutamate (Glu), norepinephrine (NE) and gamma-aminobutyric acid (GABA) converge in the PVN to influence its neuronal activity [4]. The increases of Glu and NE or decrease of GABA have been demonstrated to be involved in the control of cardiovascular reflexes [6], [7] and sympathoexcitation in HF rats [8], [9]. Recent findings showed that excess amounts of inflammatory mediators and renin-angiotensin system (RAS) components are present in the PVN and contribute to neurohumoral excitation in HF [3], [10]–[14]. As to how the inflammation factors interact with neurotransmitters, SNS and RAS in HF, our previous works in the ischemia-induced HF demonstrated the following relevant findings: (i) increased hypothalamic proinflammatory cytokines (PIC) contribute to the upregulation of central neural systems activity, including the increased SNS, central RAS and the hypothalamic-pituitary adrenal (HPA) axis activity in HF [13]; (ii) NF-κB mediates the cross-talk between RAS and PIC in the PVN in HF, and that superoxide stimulates more NF-κB in the PVN and contributes to neurohumoral excitation [12]; and (iii) increased PIC, such as brain tumor necrosis factor-α (TNF-α), modulate PVN neurotransmitters and contributes to sympathoexcitation in HF [3].

Cyclooxygenase-2 (COX-2) is the key synthetase of prostaglandin E_2_ (PGE_2_) [11], [15], a kind of ubiquitous central proinflammatory mediator, which acts in the brain and activates the hypothalamic-pituitary-adrenal (HPA) axis [16] to facilitate sympathetic drive [17] and may contribute to the pathogenesis of HF. Our recent studies suggested that activation of NF-κB in PVN is an intermediary step in the induction of COX-2 in the PVN of ischemia-induced HF rats [14], [18]. However, it is not known whether COX-2 induction results in PVN neurotransmitters and RAS diversity and further influence neuronal activity. Delgado and colleagues [19] found that COX-2 inhibitor treatment can improve left ventricular function and mortality in a murine model of doxorubicin-induced HF, but they did not investigate the potential central and neuroendocrine mechanisms for this improvement in detail. In this study, we selected the adriamycin-induced rat HF model, another widely used HF experimental model exhibiting neuroendocrine activation, for our experiments. We hypothesized that an increase in PVN COX-2 would upregulate the activities of central neural systems that contribute to increased activation of the SNS, RAS and the HPA axis in adriamycin-induced HF rats, and the protective effects of the COX-2 inhibitor, celecoxib (CLB), against adriamycin-induced HF may be involved in this mechanism.

## Results

### Mortality and Survival

During the COX-2 inhibitor treatment period (days 19 to 44), mortality was 20% (10/50) for HF+CLB treated rats versus 40% (20/50) for the HF rats. The death rate, as evaluated by chi-square criterion, was significantly higher in HF group than in the control group (*P*<0.05). CLB treatment appeared to improve survival (HF+CLB: 80%; HF: 60%) during CLB treatment period (days 19 to 44).

### Anatomical Indicators of Heart Failure

**Figure 1:**
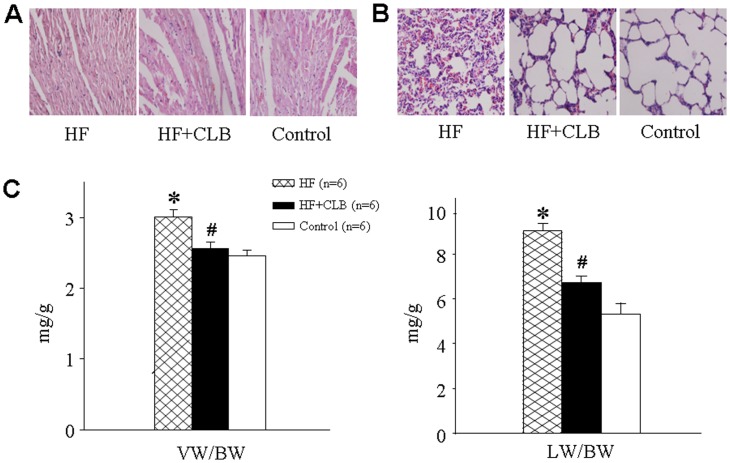
Effect of CLB on anatomical indicators of heart failure. A. Representative microphotogaph of myocardium in different groups. B. Representative microphotogaph of Lung in different groups. C. Group data showing the effect of treatment with celecoxib (CLB) *via* intragastric administration on VW/BW (ratio of ventricle to body weight) and LW/BW (ratio of lung to body weight) in ADM-induced heart failure (HF) rats. *P<0.01 *vs.* control group; ^#^ *P*<0.01 *vs.* HF group.

In HF rats, there were myocardial injury and pulmonic congestive changes, such as myocardial cells atrophy, degeneration and arranging disorder, alveolar capillary and venules proliferation and congestion. Treatment with CLB attenuated these HF-induced pathological changes (Figure 1A and 1B).

The VW/BW ratio and wet LW/BW ratio were lower in HF+CLB than HF rats (*P*<0.01, Figure 1C).

### Functional Indicators of HF

Compared with control rats, HF rats had significantly higher heart rates (HR) and left ventricular end-diastolic pressures (LVEDP), lower left ventricular peak systolic pressures (LVPSP) and maximum rate of change in left ventricular pressure (LV±dp/dtmax) (Table 1). The HF+CLB rats had significantly higher LVPSP and LV±dp/dtmax, lower HR and LVEDP than the HF rats.

**Table 1:** Effect of CLB on HR and hemodynamics in adriamycin-induced heart failure rats.

Group	HR(beats/min)	LVPSP(mmHg)	LVEDP(mmHg)	LV+dp/dtmax(mmHg/s)	LV-dp/dtmax(mmHg/s)
Control	362±23	123±7	3±2	7199±896	5608±976
HF	450±25*	84±11*	24±5*	3541±372*	3126±560*
HF+CLB	393±37^#^	112±9^#^	8±4^△#^	5598±719* ^#^	4807±627^#^

(*n* = 10). Values are mean±SEM; HR, heart rate; LVPSP, left ventricular peak systolic pressure; LVEDP, left ventricular end-diastolic pressure; LV±dp/dtmax, maximum rate of change in left ventricular pressure.

* *P*<0.01, ^△^ *P*<0.05 *vs.* control group;^ #^ *P*<0.01 *vs.* HF group.

### Plasma Levels of ANG II, NE and Atrial Natriuretic Peptide (ANP)

**Figure 2:**
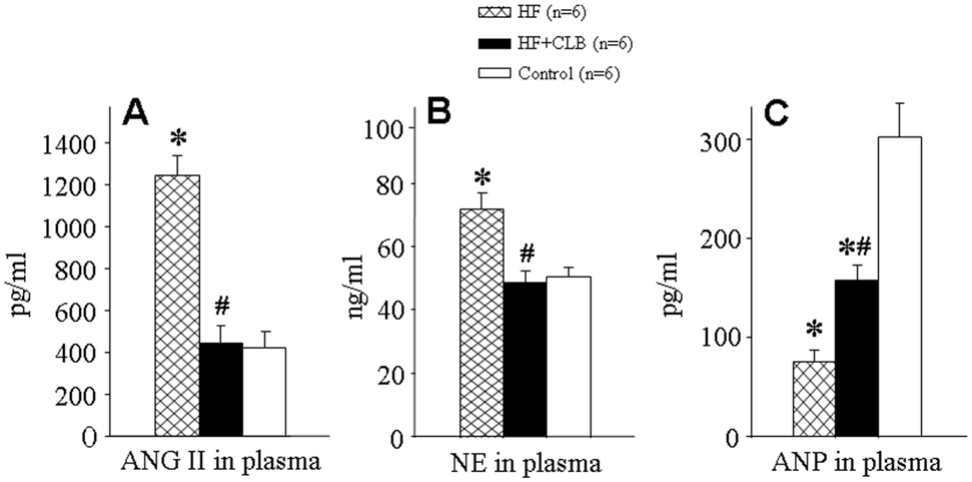
Effect of CLB on plasma levels of ANG II, NE and ANP in ADM-induced HF rats. A. Plasma ANG II. B. Plasma NE. C. Plasma ANP. HF rats had the higher plasma levels of ANG II and NE when compared with control rats, which were obviously lower in HF+CLB rats. Plasma ANP was lower in HF rats than in control rats. Plasma ANP in HF+CLB rats was significantly higher than in HF rats. *P<0.01 *vs.* control group; ^#^ *P*<0.01 *vs.* HF group.

Plasma ANG II and NE levels in HF rats were significantly higher than those in normal control rats (1248±94 versus 423±77 pg/ml for ANG II, *P*<0.01, Figure 2A; 72.11±5.26 versus 50.95±2.78 ng/ml for NE, *P*<0.01, Figure 2B). The plasma ANG II and NE levels were lower in HF+CLB rats than in HF rats (446±79 versus 1248±94 pg/ml for ANG II, *P*<0.01, Figure 2A; 48.92±3.74 versus 72.11±5.26 ng/ml for NE, *P*<0.01, Figure 2B). Plasma ANP levels were significantly lower in HF rats than in control rats (75.62±10.88 versus 303.74±46.37 pg/ml, *P*<0.01, Figure 2C). The plasma ANP level was higher in HF+CLB rats than in HF rats (159.53±33.38 versus 75.62±10.88 pg/ml, *P*<0.01, Figure 2C), but still significantly lower than in control rats (*P*<0.01).

### Myocardial Levels of ANG II and ANP

**Figure 3:**
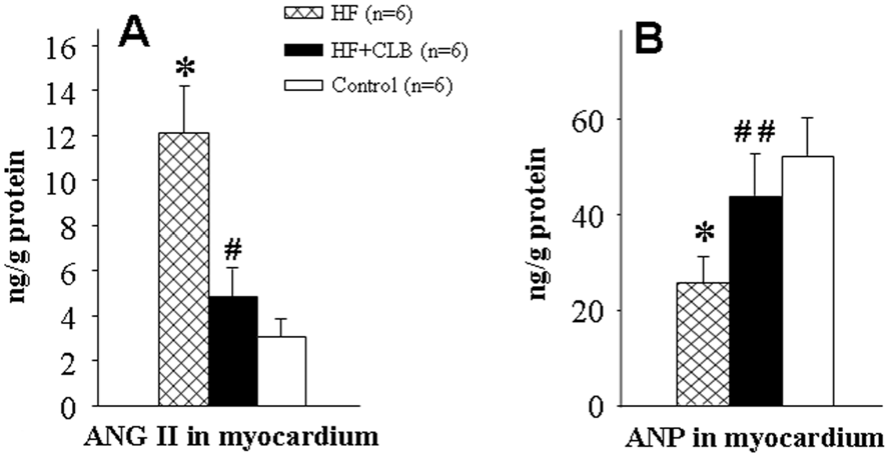
Effect of CLB on myocardial levels of ANG II and ANP in ADM-induced HF rats. A. Comparison of myocardial AngII. Myocardial ANG II level was higher in HF rats than in control rats. The myocardial ANG II level was significantly lower in HF+CLB rats than in HF rats. B. Comparison of myocardial ANP. The myocardial ANP level was lower in HF rats than in control rats. The HF+CLB rats had significantly higher ANP level than the HF model rats.*P<0.01 *vs.* control group; ^#^ *P*<0.01, ^##^ *P*<0.05 *vs.* HF group.

Changes of myocardial ANG II and ANP in HF rats were consistent with the plasma levels. Myocardial ANG II level was higher in HF rats than in control rats (12.11±2.07 versus 3.07±0.78 ng/g protein, *P*<0.01, Figure 3A). Myocardial ANG II levels were significantly lower in HF+CLB rats than in HF rats (4.87±1.22 versus 12.11±2.07 ng/g protein, *P*<0.01, Figure 3A). Myocardial ANP level was lower in HF rats than in control rats (25.69±5.43 versus 52.32±7.90 ng/g protein, *P*<0.01, Figure 3B). The HF+CLB rats had significantly higher ANP levels than the HF rats (43.84±8.72 versus 25.69±5.43 ng/g protein, *P*<0.05, Figure 3B).

### PVN Levels of ANG II and NE

**Figure 4:**
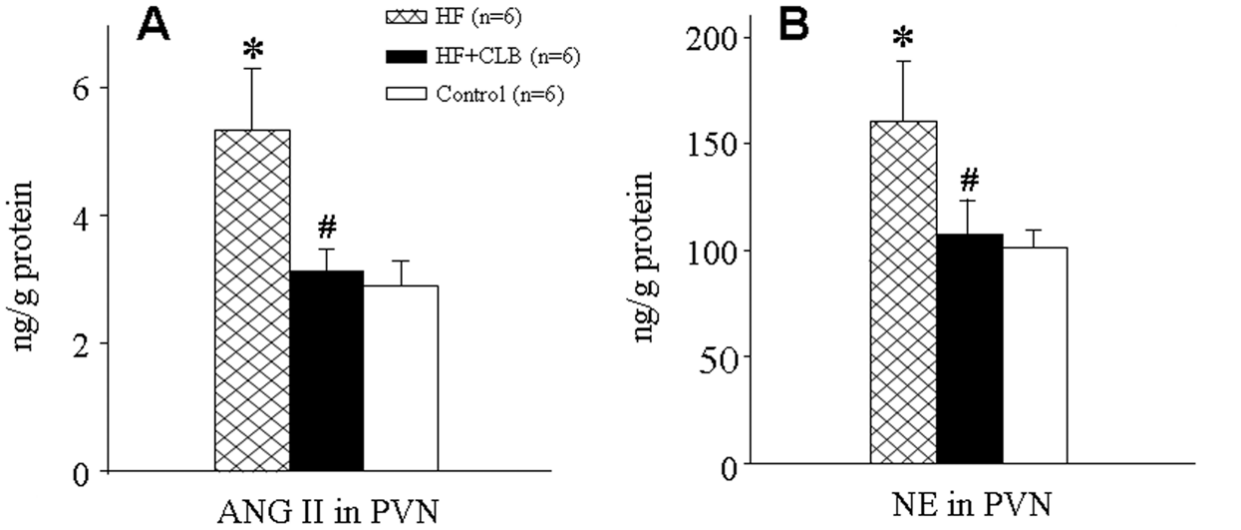
Effect of CLB on PVN levels of ANG II and NE in HF rats. A. Comparison of ANG II level. B. Comparison of NE level. The PVN levels of ANG II and NE were higher in HF rats than in control rats. The PVN levels of ANG II and NE were lower in HF+CLB rats than in HF rats. *P<0.01 *vs.* control group; ^#^ *P*<0.01 *vs.* HF group.

PVN ANG II and NE levels in HF rats were significantly higher than in control rats (5.33±0.97 versus 2.91±0.37 ng/g protein for ANG II, *P*<0.01, Figure 4A; 159.52±28.46 versus 101.06±8.01 ng/g protein for NE, P<0.01, Figure 4B). The PVN ANG II and NE levels were lower in HF+CLB rats than in HF rats (3.12±0.36 versus 5.33±0.97 ng/g protein for ANG II, P<0.01, Figure 4A; 107.54±15.82 versus 159.52±28.46 ng/g protein for NE, P<0.01, Figure 4B), and did not significantly differ from levels seen in control rats.

### Western Blot Detection of COX-2 Protein in the PVN

**Figure 5:**
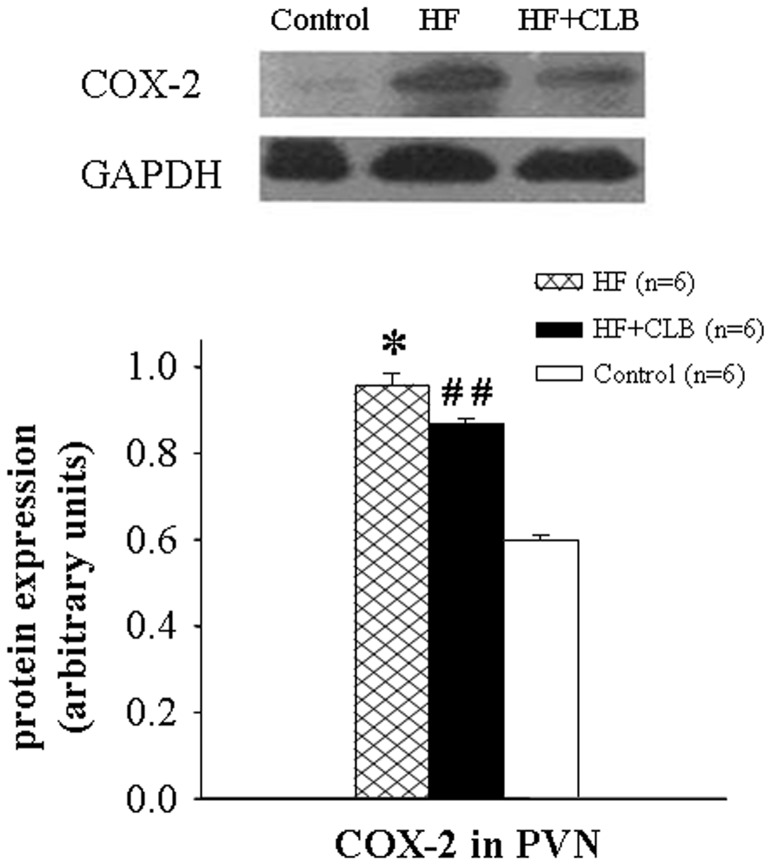
Comparison of protein expression for COX-2 in the PVN in rats. Representative Western blots were aligned with the matching grouped relative optical density values of immunoblot band. COX-2 protein expression in the PVN increased in HF rats when compared with control rats. The PVN level of COX-2 protein expression was lower in HF+CLB rats than in HF rats. *P<0.01 *vs.* control group; ^##^ *P*<0.05 *vs.* HF group.

As shown in Figure 5, HF rats had a substantial increase in COX-2 protein expression in the PVN when compared with control rats (*P*<0.01). This increase was suppressed by treatment with COX-2 inhibitor CLB (*P*<0.05).

### Immunohistochemistry for COX-2 and CRH in the PVN

**Figure 6:**
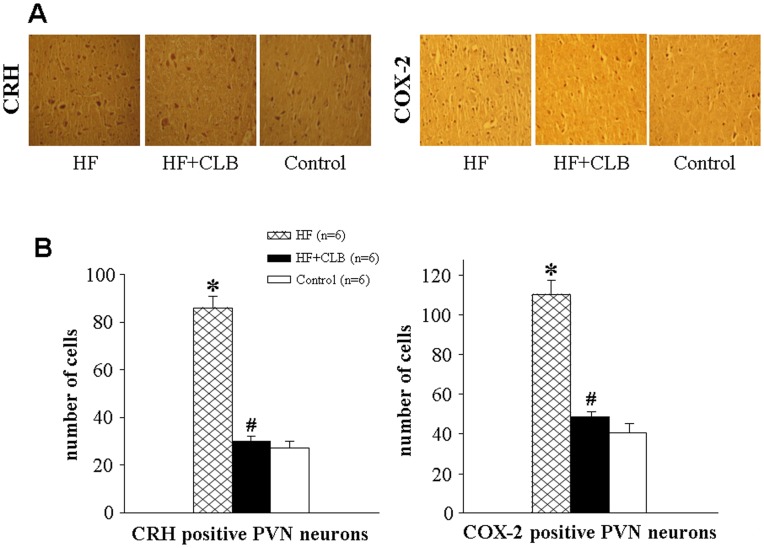
CRH and COX-2 expression in the PVN in control, HF and HF+CLB rats. A. Immunohistochemistry for CRH (deep brown dots) and COX-2 (brown) positive neurons in control, HF and HF+CLB rats. B. Bar graph comparing numbers of PVN CRH and COX-2 positive neurons in different groups. The HF rats had more COX-2 and CRH-positive neurons in the PVN than control rats. There were fewer positive neurons of COX-2 and CRH in the PVN of HF+CLB rats than in HF rats. *P<0.01 *vs.* control group; ^#^ *P*<0.01 *vs.* HF group.

Immunohistochemistry studies revealed that HF rats had more COX-2 and CRH-positive neurons in the PVN than control rats (Figure 6). There were fewer positive COX-2 and CRH neurons in the PVN of HF+CLB rats than in HF rats (Figure 6).

### Neurotransmitters in the PVN

**Figure 7:**
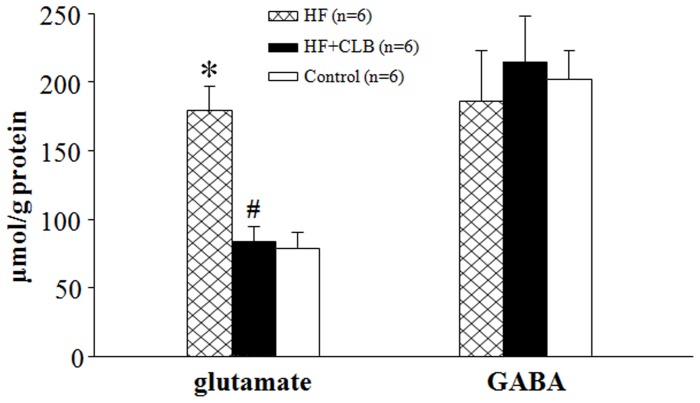
Glutamate and GABA release in the PVN in control, HF and HF+CLB rats. Compared with control rats, HF rats had higher levels of glutamate in the PVN. Glutamate release in the PVN was lower in HF+CLB rats than in HF rats. However, there were no significant differences in GABA among control, HF, and HF+CLB rats. *P<0.01 *vs.* control group; ^#^ *P*<0.01 *vs.* HF group.

Compared with control rats, HF rats had higher levels of glutamate in the PVN (179±18 versus 79±11 µmol/g protein, *P*<0.01, Figure 7). Glutamate in the PVN in HF+CLB rats was decreased after 4 weeks’ treatment (83±12 versus 179±18 µmol/g protein, *P*<0.01, Figure 7), and levels did not differ significantly from those seen in control rats. There were no significant differences detected in GABA levels among any of the rat groups.

## Discussion

The novel findings of the present study are: (i) adriamycin-induced HF rats had higher PVN levels of glutamate, NE, ANG II, CRH and COX-2, higher plasma levels of ANG II and NE, and lower levels of ANP in plasma and myocardium; and (ii) treatment with CLB attenuated these HF-induced changes, reduced mortality and improved myocardial atrophy, pulmonary congestion and hemodynamic parameters of HF rats. Our findings suggest that COX-2 might be an important factor responsible for the PVN activation and contributes to sympathoexcitation and RAS activation in adriamycin-induced heart failure.

Neuroendocrine activation is believed to play an important role in the pathogenesis of chronic congestive heart failure. Specifically, excess activation of SNS, RAS and the HPA axis have been implicated [13], [20]–[24]. Intraperitoneal injection of a cumulative dose of adriamycin over a period of 2 to 6 weeks has been shown to induce congestive heart failure and neuroendocrine activation [19], [25]–[29]. The present study found that PVN COX-2 is implicated in the regulation of neurohormones in adriamycin-induced HF rats *via* its influence on sympathetic drive, and *via* activation of RAS and HPA axis. It is demonstrated that PGE_2_, as the principal product of COX-2 in the hypothalamus, plays a major role in activating the HPA axis [16]. Further, upregulation of the brain RAS results in sympathoexcitation in HF [10], [30]–[32], and ANG II in hypothalamus may modulate thirst and salt appetite [33], [34] and activate neurons containing CRH [35]–[37] and arginine vasopressin [38], [39]. Our previous studies have also demonstrated that increased hypothalamic proinflammatory cytokines activate RAS and the HPA axis [13], modulate PVN neurotransmitters, and contribute to sympathoexcitation in ischemia-induced HF [3].

In summary, the findings from the present study suggest that COX-2 in the PVN may be an intermediary step for PVN neuronal activation and excitatory neurotransmitter release, which further contributes to sympathoexcitation and RAS activation in adriamycin-induced heart failure. Treatment with a COX-2 inhibitor attenuates sympathoexcitation and RAS activation in adriamycin-induced heart failure.

## Materials and Methods

### Drugs and Reagents

The COX-2 inhibitor celecoxib (CLB) used in this study was obtained from Pfizer Pharmaceuticals Ltd (Newyork, USA). Adriamycin (ADM, Doxorubicin) was purchased from a local pharmacy. O-phthaldialdehyde (OPA) and standard preparation of L-glutamate and GABA were purchased from Sigma-Aldrich (St. Louis, MO, USA). Rat ANG II, NE and ANP ELISA Kits were offered by Cusabio Biotech. The goat anti-COX-2 monoclonal antibody, rabbit anti-CRH polyclonal antibody, anti-GAPDH antibodies were purchased from Santa Cruz Biotechnology. Streptavidin/Peroxidase Histostain-Plus Kit was obtained from ZYMED (USA). BCA Total Protein Quantification Kit, SDS-PAGE gel Kit and other Western-blotting regents were purchased from Beyotime Institute of Biotechnology (Shanghai, China).

### Animal Preparation

Adult male Sprague-Dawley rats weighing 220 to 250 g were provided by Experimental Animal Center of Shantou University Medical College. These studies were performed in accordance with the “Guiding principles for research involving animals and human beings” [40]. All experimental protocols were approved by the Review Committee for the Use of Human or Animal Subjects of Shantou University Medical College.

### Heart Failure Induction Protocol

Heart failure was induced experimentally by intraperitoneal injections of the cardiotoxic agent adriamycin (ADM) for a cumulative dose of 15 mg/kg in 6 injections (ADM, 2.5 mg/kg×6) over a 2 week period. This reproducible model described as previously could induce progression to end-stage heart failure at most states [25]–[27]. Animals in the control group were treated with saline. The weight of rat was measured to recalculate dose prior to every administration, and was kept on monitoring once a week subsequently until the termination of test.

### COX-2 Inhibitor Management Protocol

On the day 19 of the HF induction protocol, 100 HF-induced rats were randomly divided into 2 groups, one to receive the COX-2 inhibitor CLB (i.e. HF+CLB, n = 50) and the other to serve as HF group (n = 50). The COX-2 inhibitor celecoxib (CLB) we used is a selective inhibitor of COX-2 that has no significant activity against COX-1 when administered in clinical effective concentrations. In the HF+CLB group, rats were treated with CLB by intragastric administration (3 mg/kg/day). In the normal control and HF group, the rats were treated with equal volume saline. On the 44th day of the experiment, rats were hemodynamically evaluated and killed to be assayed in morphology and biochemistry.

### Electrophysiological Recordings

The electrophysiological recordings were performed as previously described [3], [12], [13] by BL-820S Data Acquisition & Analysis System analyzing system (Chengdu TME Technology Co, Ltd, Chengdu, China). At the end of experiment, rats were anesthetized (pentobarbital, 40 mg/kg i.p). Electrocardiogram (ECG) was recorded before hemodynamics assessment and heart rate (HR) was derived from ECG tracing. A micromanometer connected with PE-50 catheter (Millar Instruments) was inserted into the left ventricle via the right carotid artery. LVPSP, LVEDP and LV±dp/dtmax were derived from the LV pressure tracing.

### Preparation of Blood and Tissue Samples

Rats were decapitated while still under deep anaesthesia to collect trunk blood and tissue samples. Trunk blood was collected in chilled ethylene diamine tetra acetic acid tubes. Plasma samples were separated and stored at −86°C until assayed for ANG II, NE and ANP levels. The brain, heart and lung were harvested and weighed with 30 seconds, rapidly frozen in liquid nitrogen, and used for histopathological examination and for biochemical assay as described below.

Palkovits’s microdissection method was applied to isolate the PVN as described before [3], [41]. In brief, the brain was sectioned serially in 300 mm increments from the bregma to lambda using a cryostat. The sections were transferred to coverslips and the PVN was punched with the help of a stereotaxic atlas and stored at −86°C. Frozen heart and PVN tissue samples were homogenized in PBS containing a protease inhibitor PMSF and then centrifuged at 3500 rpm. The resulting supernatants were assayed for protein concentration by the bicinchoninic acid method with a BCA Protein Assay kit and then subjected to further analyses for ELISA, HPLC and Western blot.

### Anatomic Measurements

Both lung weight (LW) and heart ventricular weight (VW) versus body weight (BW) were used as indexes of pulmonary congestion and ventricular remodeling, generally considered as two indices of the HF severity.

### ANG II, NE and ANP Assessments in Plasma and Tissues

ANG II, NE and ANP levels in plasma and tissues were measured using rat ELISA kits according to the manufacturer’s specifications. The minimum detectable concentration of ANG II, NE and ANP was 0.45, 4.7 and 15.6 pg/ml respectively.

### Immunohistochemical Studies

A usual avidin-biotin-peroxidase complex procedure was applied [42]. Transverse sections from brains were obtained from the region approximately 1.80 mm from the bregma. Immunohistochemical labelling was performed in floating sections as described previously [43] to identify COX-2 (Santa Cruz Biotechnology) and corticotropin releasing hormone (CRH; Santa Cruz Biotechnology). Images were captured at ×200 magnification using a BX-40 Olympus microscope. For each rat, tagged neurons within the bilateral borders of PVN were counted manually in 3 adjacent coronal transverse sections, and an average value was reported (positive neurons/100 µm^2^). BI-2000 Medical Image Analysis System (Chengdu TME Technology, Chengdu, China) was used to confirm the manual cell counts and to quantify the intensity of COX-2 expression in the PVN.

### Western Blot

Protein extracted from punches of the PVN was used for measurement of COX-2 and CRH expression by western blotting as described by others [44]. Protein loading was controlled by probing all western blots with anti-GAPDH antibody and normalizing COX-2 and CRH protein intensities to that of GAPDH. Band densities were analyzed with NIH ImageJ software.

### Measurements of Glutamate and GABA in PVN Tissues

The contents of glutamate (Glu) and GABA were assayed by high performance liquid chromatography (HPLC) with fluorescence detection using a spectrophotometer (Varian, Holland) as described previously [45]. The derivatization of detected specimens was performed in an automatic sampler (Prostar, Varian, Holland). KR100 -5C18 column (Kromasil, Akzonobel, Switzerland) were used to dissociate the derivatives. Buffer A (0.1 M PBS, pH6.8) and Buffer B (pure methanol) constituted the gradient elution mobile phase. Data were analyzed with the ANASTAR software. External standard method was applied to quantitate the concentrations of Glu and GABA.

### Statistical Analysis

Results are presented as mean±SEM. Statistical comparisons were analyzed by using one-way analysis of variance (ANOVA) and Fisher Least-significant difference test. A probability of *P*<0.05 was considered to be statistically significant.
